# Two kinds of traditional Chinese medicine prescriptions reduce thymic inflammation levels and improve humoral immunity of finishing pigs

**DOI:** 10.3389/fvets.2022.929112

**Published:** 2022-09-06

**Authors:** Xiaoyu Wang, Jiajia Chen, Fan Yang, Farah Ali, Yaqin Mao, Aiming Hu, Tianfang Xu, Yan Yang, Feibing Wang, Guangbin Zhou, Xiaowang Guo, Huabin Cao

**Affiliations:** ^1^Jiangxi Provincial Key Laboratory for Animal Health, College of Animal Science and Technology, Institute of Animal Population Health, Jiangxi Agricultural University, Nanchang, China; ^2^Department of Animal Science and Technology, Jiangxi Biotech Vocational College, Nanchang, China; ^3^Department of Theriogenology, Faculty of Veterinary & Animal Sciences, The Islamia University of Bahawalpur, Pakistan, Bahawalpur, Pakistan; ^4^China Institute of Veterinary Drug Control, MOA Center for Veterinary Drug Evaluation, Beijing, China; ^5^Jian City Livestock and Veterinary Bureau, Jiangxi, China; ^6^Jiangxi Agricultural Technology Extension Center, Nanchang, China; ^7^Agricultural Technology Extension Center, Jinxi County Agriculture and Rural Bureau, Fuzhou, China; ^8^Animal Epidemic Prevention and Quarantine Unit, Fengcheng Agricultural and Rural Bureau, Fengcheng, China; ^9^Yichun Agriculture and Rural Affairs Bureau, Yichun, China

**Keywords:** traditional Chinese medicine, finishing pigs, immune function, inflammation, thymus

## Abstract

In animal husbandry, traditional Chinese medicine (TCM) as a reasonable alternative to antibiotics has attracted more and more concerns to reduce microbial resistance. This study was aimed to investigate the effects of dietary supplementation with TCM prescriptions on serum parameters and thymus inflammation responses in finishing pigs. Thirty finishing pigs were randomly divided into three groups, which included the Con group (basal diet), the TCM1 group (basal diet supplemented with *Xiao Jian Zhong* prescriptions), and the TCM2 group (basal diet supplemented with *Jingsananli*-sepsis). The results showed that the contents of C3 and C4 in the serum were significantly increased in both the TCM1 and TCM2 groups compared to the Con group on day 30. Similarly, the levels of IgA, IgG, and IgM were increased in the TCM2 group, and only the level of IgM in TCM1 was increased on day 30. Meanwhile, the levels of classical swine fever virus (CSFV) and respiratory syndrome virus (PRRSV) antibodies had a notable increase in the TCM1 and TCM2 groups. Both TCM1 and TCM2 inhibited the levels of TLR4/MyD88/NF-κB signaling pathway-related mRNA (TLR4, MyD88, NF-κB, IL6, IL8, and TNF-α) and protein (p-IκBα and p-P65) expression levels in the thymus. In conclusion, dietary supplementation with TCM could reduce thymic inflammation levels and improve humoral immunity of finishing pigs.

## Introduction

As we all know, piglet breeding is the most critical period in the process of pig breeding. To safeguard the health of finishing pigs during the nursery period, the use of antimicrobials either in feed or in water has been an essential tool. Antibiotics are used in swine feed as a growth promoter, to improve feed efficiency, and to reduce susceptibility to bacterial infections (1, 2). Nevertheless, abuse of antibiotic feed additives not only leads to excessive antibiotic residues in animals but also endangers human food safety and health. The China Ministry of Agriculture (MOA) gradually reduced the types and doses of antibiotics allowed in feeds in 2017 with the intent to ban the usage of antibiotics in feed additives by 2020 (3). Thus, it is necessary to develop new alternatives instead of using antibiotics to control diseases.

Traditional Chinese medicine (TCM) has lately received greater attention from researchers because of its natural structure and biological activity that can enhance or restore the immune system (4, 5). A recent study has shown that some phytochemicals were beneficial to health by promoting immune function and reducing inflammation responses (6). According to the theory of TCM, prescriptions are usually composed of several herbs or minerals, one of which represents the principal component and the other serves as an auxiliary component to assist or promote the transmission of the principal component. In most cases, the mixture of a variety of TCM will have a synergistic therapeutic effect (7).

The TLR4/MyD88/NF-κB pathway is a classical toll-like receptor activation pathway that plays an essential role in anti-inflammatory and immune regulations. A large number of studies have reported that inhibition of the TLR4/MyD88/NF-κB pathway could effectively inhibit the activation of NF-κB and reduce the secretion of pro-inflammatory factors such as TNF-a and IL-6, thereby inhibiting the occurrence of inflammation (8, 9). A previous study demonstrated that cinnamaldehyde has anti-inflammatory effects that can prevent inflammation-related health problems associated with over-activation of the TLR4 signaling pathway (10). Meanwhile, Luo et al. (11) found that pachymaran enhanced the immune function of mice by regulating genes associated with T and B cell functions. Additionally, it is reported that saikosaponin-a strongly inhibited pro-inflammatory mediators (12). Thus, dietary supplementation with TCM may be a feasible strategy to replace or reduce the use of antibiotics by enhancing immune responses, consequently promoting growth performance in finishing pigs (13). In conclusion, natural plants with pharmacological activities are recommended as dietary supplements or therapeutic agents to effectively care for the organism.

There is little research on the effects of TCM prescriptions on the immunity of finishing pigs. Therefore, this study was conducted to assess the effects of TCM1 and TCM2 on serum parameters and thymus inflammation responses in finishing pigs, and aimed to provide valuable insights for improving the immune performance of finishing pigs.

## Materials and methods

### Animals and treatments

This trial was performed in the Researching and Teaching Base of Jiangxi Agricultural University and treated for 60 days. Thirty finishing pigs (white × landrace × Duroc) weighing 21.43 ± 2.86 kg were allowed *ad libitum* access to feed and water throughout the experimental period. Experimental animals of all three treatment groups were fed with the same basal diet, which was formulated to meet the nutrient requirements of finishing pigs (14). The composition and nutrient levels of basal diet are shown in Table 1. The finishing pigs were randomly allotted to three treatment groups (*n* = 10): The Con group (basal diet), the TCM1 group (basal diet + 10 g/kg *Xiao Jian Zhong*, XJZ) and the TCM2 group (basal diet + 3 g/kg *Jingsananli*-sepsis, JSS). The doses of TCM1 and TCM2 were evaluated according to our preliminary study and traditional Chinese pharmacopeia (2005). All raw materials of TCM1 were bought from Chinese Traditional Medicine Chang Sheng (Jiangxi, China), and TCM2 was provided by Jiangxi Chuang Dao Animal Health Products Co., Ltd. (Jiangxi, China). The composition and main active constituents of TCM1 and TCM2 are presented in Table 2. All experimental protocols were approved by the Committee for the Care and Use of Experimental Animals, Jiangxi Agricultural University, Jiangxi, China.

**Table 1:** Composition and nutrient levels of basal diets (air-dry basis)*^a^*.

**Ingredients**	**Content (%)**	**Analyzed composition, g/kg**	**Content (%)**
Maize	55.80	DM	89.21
Soybean meal	16.3	DE (MJ/kg)	14.36
Fermented soybean meal	7.0	CP	19.63
Wheat middling	4.5	Lysine	1.32
Fish meal	2.5	Methionine	0.43
Dried porcine solubles	2.5	Methionine+Cystine	0.77
Whey powder	6.25	Threonine	0.81
Soy oil	1.65	Calcium	0.96
Lysine	0.25	Total phosphorus	0.60
Methionine	0.1		
Limestone	1.05		
CaHPH_4_	0.80		
NaCl	0.30		
premix	1.00		

a The premix provides the following per kilogram diet:Vitamin A 8 000 IU, Vitamin D 2 500 IU, Vitamin E 15 mg, nicotinic acid 20 mg, D-pantothenie 10 mg, riboflavin 4 mg, biotin 0.06 mg, folic acid 0.2 mg, thiamine 2mg, choline chloride 500 mg, Cu 165 mg, Fe 110 mg, Mn 80 mg, Zn 330 mg, Se 0.20 mg.

**Table 2:** Composition and main active constituents of TCM1 and TCM2 (air dry basis)*^a^*.

**Latin name**	**Main active constituent**	**Used part**	**Content (%)**
**TCM1**			
*Cassia Twig*	*Cinnamaldehyde*	Dried twig	13
*Glycyrrhiza uralensis*	*Glycyrrhizin*	Dried root	4
*Ziziphus zizyphus*	*Jujuba polysaccharide*	Dried fructification	4
*Cynanchum otophyllum*	*Paeoniflorin*	Dried root	13
*Zingiber officinale Roscoe*	*Ginger oleoresin*	Dried root	6
*Rhizoma Atractylodes*	*Atractylodine*	Dried root	14
*Atractylodes macrocephala*	*Biatractylolide*	Dried root	10.5
*Poria cocos*	*Pachymaran*	Dried sclerotium	10.5
*Coptis chinensis Franch*.	*Berberine*	Dried root	4
Maltose	Maltose	-	21
Total			100
**TCM2**			
*Nepeta cataria L*.	*Nepeta Cataria Oil*	Dried stem	16.5
*Radix Saposhnikoviae*	*Chromone glycoside*	Dried root	16.5
*Notopterygium incisum*	*Notopterol*	Dried root and stem	16.5
*Radix Angelicae*	*Heraclenin*	Dried root	16.5
*Radix bupleuri*	*Saikosaponin*	Dried root	10
*Radix Peucedani*	*peucedanin*	Dried root	10
*Poria cocos*	*Pachymaran*	Dried sclerotium	10
*Glycyrrhiza uralensis*	*Glycyrrhizin*	Dried root	4
Total			100

a Main active constituents of TCM come from Chinese pharmacopeia (2005).

### Sample collection

The experimental period lasted 60 days, and blood samples were collected on days 0, 30, and 60 and centrifuged (3,000 × g, 10 min) to obtain the serum which was then stored at −20°C for follow-up studies. All the finishing pigs were killed by euthanasia with an intravenous injection of sodium pentobarbital (40 mg/kg body weight), and the thymus of the finishing pigs was collected immediately after euthanasia, rapidly frozen in liquid nitrogen, and stored at −80°C for various analyses on day 60.

### Serum parameter analysis

IgA, IgM, IgG, C3, and C4 were assayed following the manufacturer's instructions and using porcine-specific immunoturbidimetry kits (Nanjing Jiancheng Bioengineering Institute, China).

### Blocking enzyme-linked immunosorbent assay (ELISA)

The serum samples were assayed following the manufacturer's instructions and using the IDEXX classical swine fever virus (CSFV) antibody test kit and IDEXX porcine reproductive and respiratory syndrome virus (PRRSV) antibody test kit (IDEXX Laboratories, Netherlands).

### Real-time quantitative polymerase chain reaction (qRT-PCR)

The mRNA transcription levels of TLR4/MyD88/NF-κB signal pathway-related genes (TLR4, MyD88, NF-κB, IL-6, IL-8, and TNF-α) in the thymus were determined by qRT-PCR assay according to the manufacturer's instructions. Briefly, total RNA was isolated using the TransZol Up Reagent (TransGen Biotech, Beijing, China). Then, cDNA was synthesized using a TransScript® One-Step gDNA Removal and cDNA Synthesis SuperMix reagent kit (TransGen Biotech, Beijing, China) according to the kit's instructions and stored at −20°C for SYBR Green qRT-PCR. Gene-specific primers of all the genes were designed using the Primer Premier software (PREMIER Biosoft International, CA, United States). The GAPDH gene was used as an internal reference, and the primer sequences are shown in Table 3. The qRT-PCR profiles were as follows: 95°C for 10 min, 40 cycles at 95°C for 15 s, 60°C for 60 s, and extension at 95°C for 15 s. All the reactions were carried out using an ABI 7900HT machine (Applied Biosystems, United States).

**Table 3:** Primers used in this study.

**Target**	**Gene bank number**	**Primers sequences (5'-3')**
TNF-α	NM_214022.1	F: CCAATGGGCAGAHTGGGTATG
		R: TGAAGAGGACCTGGGAGTAG
IL-6	NM_001252429.1	F: TGGCTACTGCCTTCCCTACC
		R: CAGAGATTTTGCCGAGGATG
IL-8	NM_213867.1	F: TTCGATGCCAGTGCATAAATA
		R: CTGTACAACCTTCTGCACCCA
GAPDH	NM_001206359	F: ACTCACTCTTCCACTTTTGATGCT
		R: TGTTGCTGTAGCCAAATTCA
MyD88	NM_001099923.1	F: TGGTAGTGGTTGTCTCTGATGA
		R: TGGAGAGAGGCTGAGTGCAA
NF-κB	NM_001048232.1	F: CTCGCACAAGGAGACATGAA
		R: ACTCAGCCGGAAGGCATTAT
TLR4	NM_001113039.1	F: GCCATCGCTGCTAACATCATC
		R: CTCATACTCAAAGATACACCATCGG

### Western blot

The total protein from about 0.1g thymus of each piglet was extracted using a RIPA lysis buffer (APPLYGEN, Beijing, China) supplemented with 1 mM phenylmethanesulfonyl fluoride and phosphatase inhibitor, and the concentrations of total proteins were measured using a BCA (bicinchoninic acid) protein assay kit (Solarbio, China). Protein supernatant was separated by 10% SDS-PAGE and transferred into PVDF membranes. After blocking with 5% skimmed milk powder, the membranes were incubated with appropriate primary antibodies overnight at 4°C, followed by incubation with corresponding secondary antibodies for 1 h at room temperature. The membranes were washed thrice for 10 min each, incubated with a SuperSignal chemiluminescent substrate (Pierce), and imaged with ChemiDoc XRS+ Imaging System (Bio-Rad). Blots were semi-quantified using the ImageJ software. Primary antibodies for NF-κB p65 (Bioss, 1:1,000) and p-NF-κB p65, p-IκB-α (Bioss, 1:800) and phospho-IκB-α were used in this study.

### Statistical analysis

A statistical analysis was performed using the SPSS 17.0 software (SPSS Inc., Chicago, United States). All the results are expressed in the format of mean ± standard deviation (SD). Comparisons between two or multiple groups were made by the Student's *t*-test or one-way ANOVA. A *P*-value of <0.05 was significant.

## Results

### Effects of TCM1 and TCM2 on immunoglobulins in serum

**Figure 1:**
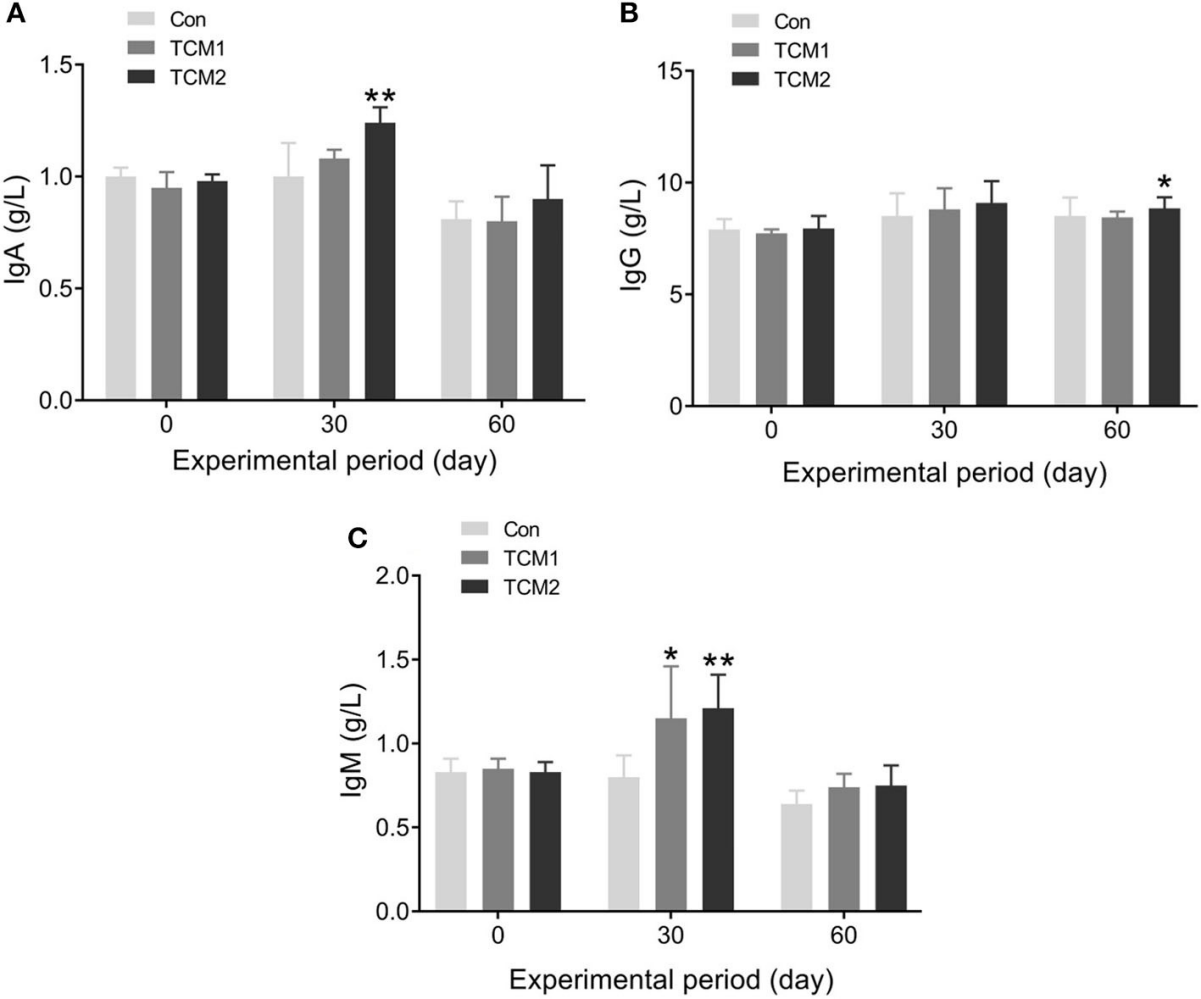
Effect of TCM1 and TCM2 on serums IgA, IgG, and IgM in finishing swine. “*” indicates significant difference compared with the control group (*P < 0.05, **P < 0.01). Below is the same. **(A)** IgA level. **(B)** IgG level. **(C)** IgM level.

The effects of TCM1 and TCM2 on serum immunoglobulins are shown in Figure 1. Compared to the Con group, the TCM2 group showed a higher concentration of IgA (*P* < 0.01) on day 30 and a higher concentration of IgG (*P* < 0.05) on day 60. Furthermore, the TCM1 group showed higher serum levels of IgM (*P* < 0.05) on day 30, and the TCM2 group showed a higher concentration of IgM (*P* < 0.01) on day 30.

### Effects of TCM1 and TCM2 on complement in serum

**Figure 2:**
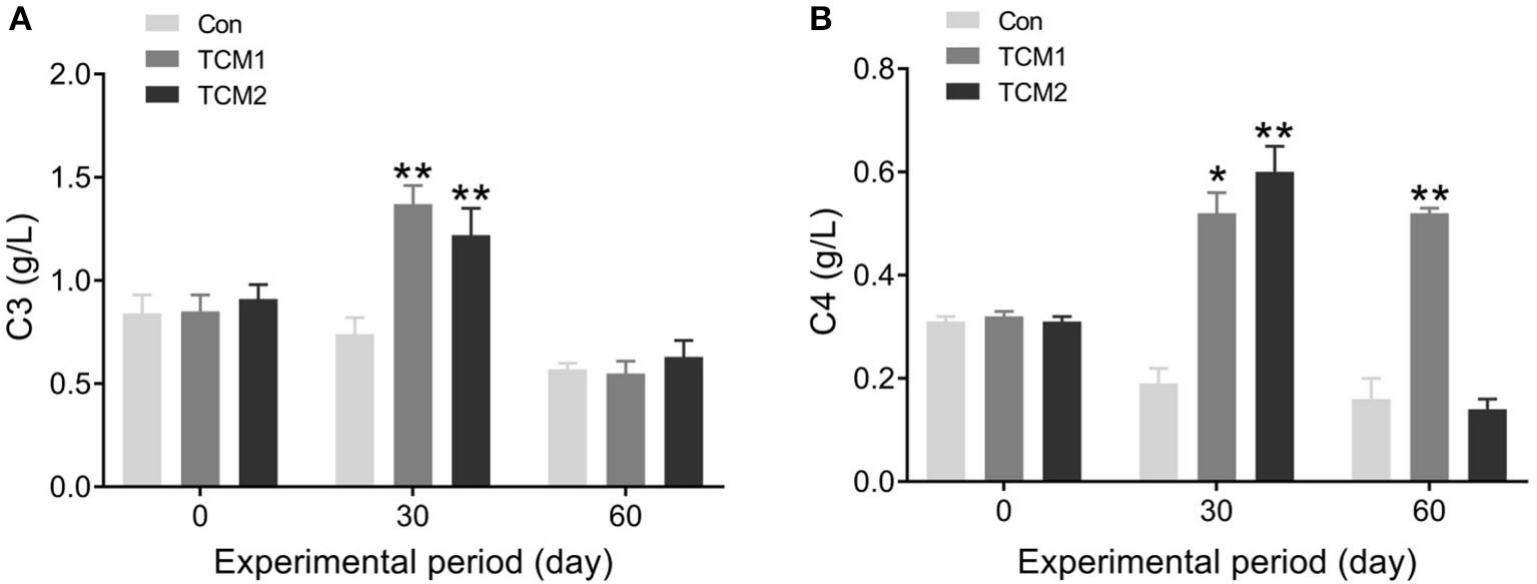
Effect of TCM1 and TCM2 on the levels of serums C3 and C4 in finishing swine. **(A)** The serum C3 level. **(B)** The serum C4 level. “*” indicates significant difference compared with control group (*P < 0.05 and **P < 0.01).

The effects of TCM1 and TCM2 on serum complement are shown in Figure 2. Compared to the Con group, the TCM1 and TCM2 groups showed the highest concentration of C3 (*P* < 0.01) on day 30. No dramatic difference was observed in the serum concentrations of C3 among the three groups on day 60. The TCM2 group showed the highest concentration of C4 (*P* < 0.01) on day 30, and the TCM1 group showed a higher concentration of C4 (*P* < 0.05) on day 30. Furthermore, the TCM1 group showed highest serum levels of C4 (*P* < 0.01) on day 60.

### CSFV and PRRSV antibodies in finishing pigs

**Figure 3:**
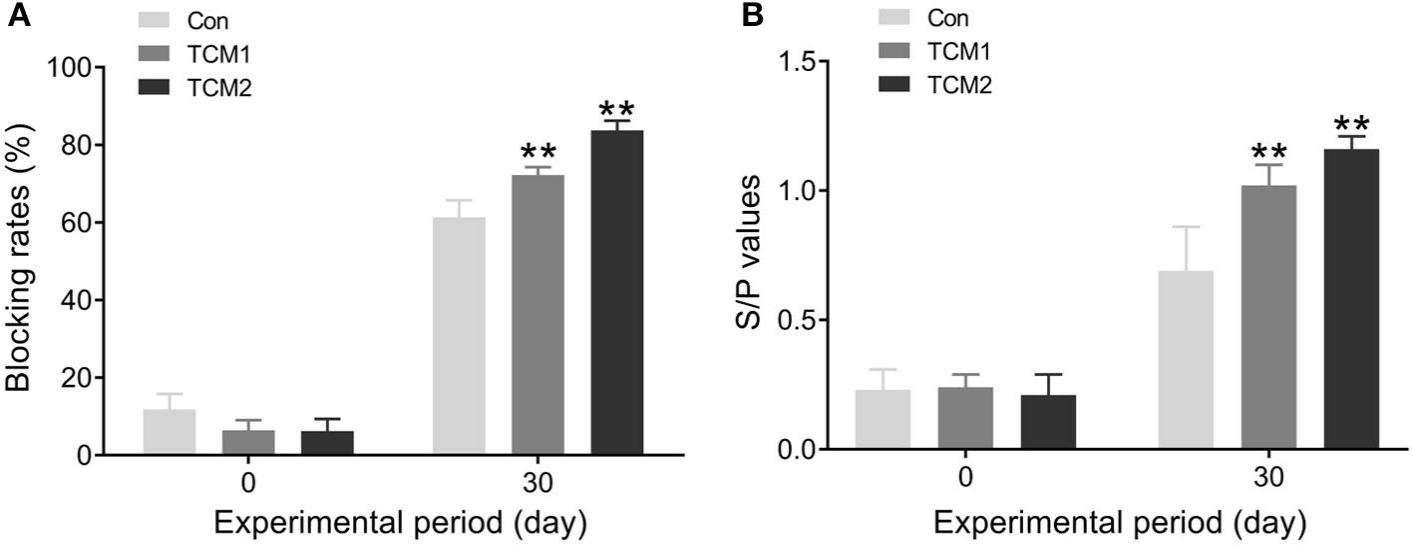
Effect of TCM1 and TCM2 on CSFV and PRRSV antibodies in finishing swine. **(A)** The concentration of CSFV antibody. **(B)** The concentration of PRRSV antibody. “*” indicates a significant difference compared with control group (*P < 0.05 and **P < 0.01).

CSFV and PRRSV antibodies in the finishing pigs are evaluated in Figure 3. Compared to the Con group, the TCM1 and TCM2 groups showed the highest concentration of CSFV and PRRSV antibodies (*P* < 0.01) on day 30.

### Effects of TCM1 and TCM2 on TLR4/MyD88/NF-κB pathway-related mRNA and protein levels

**Figure 4:**
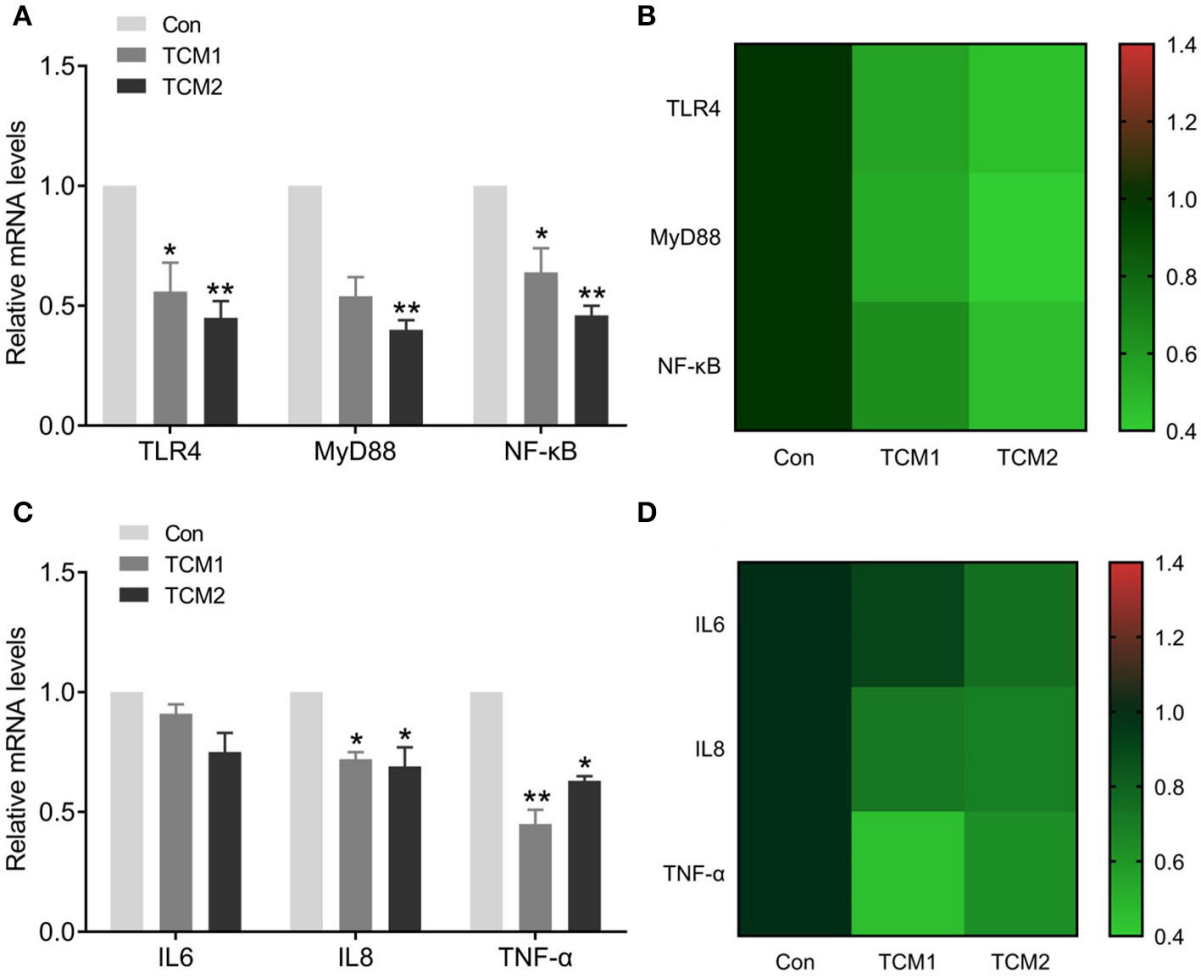
Effects of TCM supplement on mRNA levels of the TLR4/MyD88/NF-κB signaling pathway and inflammatory cytokine genes. **(A)** Effects of TCM supplement on mRNA levels of TLR4/MyD88/NF-κB pathway-related genes. **(B)** Heat map shows the mRNA levels of TLR4/MyD88/NF-κB signaling pathway-related genes. **(C)** Effects of TCM supplement on mRNA levels of inflammatory cytokine genes. **(D)** Heat map shows the mRNA levels of inflammatory cytokines genes. “*” indicates a significant difference compared with control group (*P < 0.05 and **P < 0.01).

**Figure 5:**
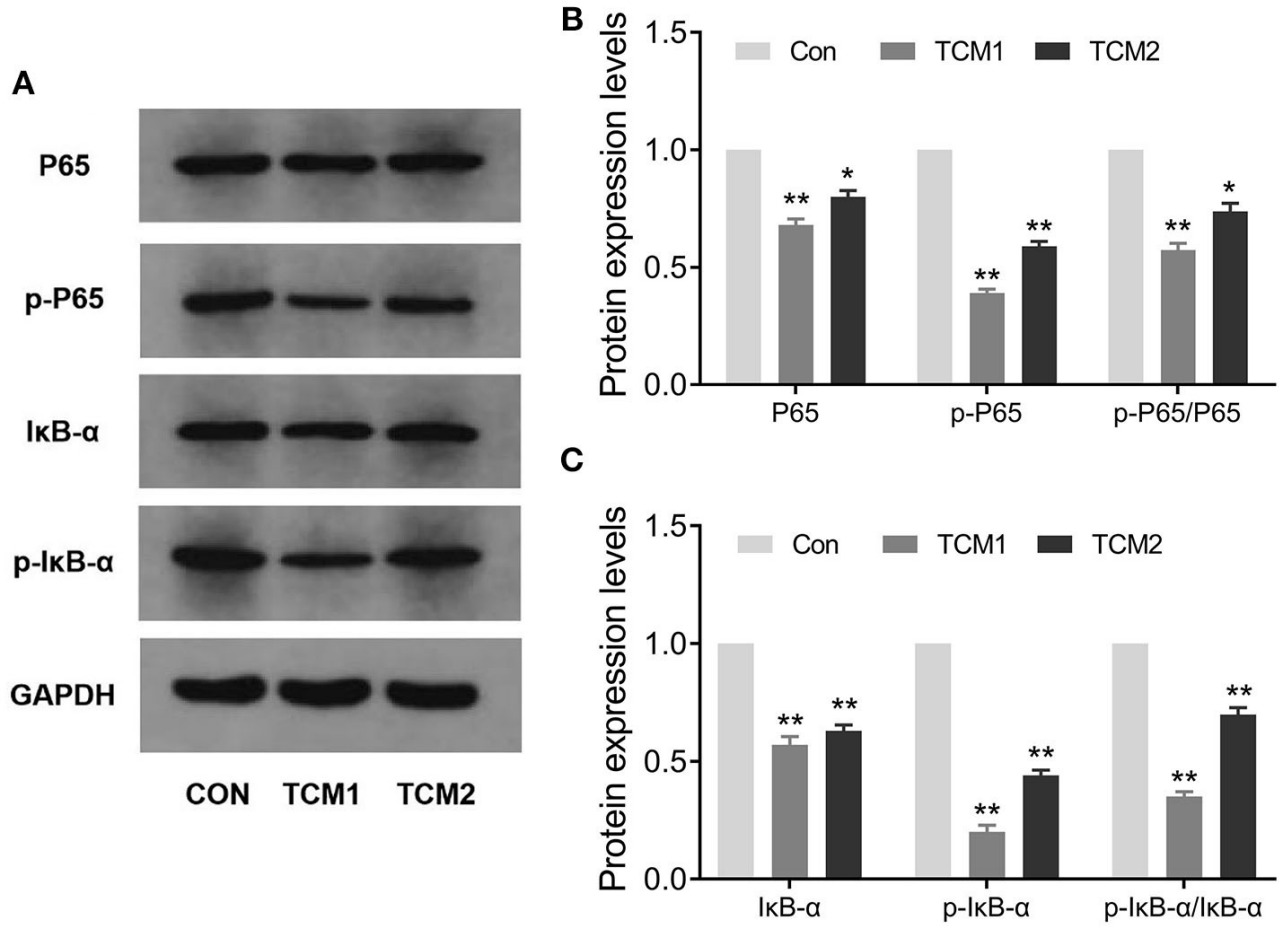
Effects of TCM supplement on key proteins levels of the NF-κB signaling pathway. **(A,B)** Immunoblot analysis of P65 and p-P65 proteins. **(C)** Immunoblot analysis of IκBα and p-IκBα proteins. “*” indicates a significant difference compared with control group (*P < 0.05 and **P < 0.01).

The effects of TCM1 and TCM2 treatments on mRNA expression levels of the TLR4/MyD88/NF-κB signaling pathway in thymus tissues of the finishing pigs are shown in Figures 4A,B. Compared to the Con group, the mRNA expression levels of MyD88 were decreased in the TCM1 and TCM2 (*P* < 0.01) treatments; NF–κB levels in all the treatments group were decreased and significant in TCM1 (*P* < 0.05) and TCM2 (*P* < 0.01). Similarly, the levels of TLR4 were downregulated in the TCM1 (*P* < 0.05) and TCM2 (*P* < 0.01) treatments. The protein levels of P65, p-P65, IκBα, and p-IκBα were notably increased (*P* < 0.01 or *P* < 0.001) in the TCM1 and TCM2 groups (Figures 5A–C). Additionally, the p-P65/P65 ratio and the p-IκBα/IκBα ratio in both TCM1 and TCM2 groups was down-regulated (*P* < 0.01 or *P* < 0.001) in comparison with the control group.

### Effects of TCM1 and TCM2 on inflammatory cytokine mRNA levels

The effects of TCM supplementation on thymus tissue mRNA expression levels of IL-6, IL-8 and TNF-α are shown in Figures 4C,D. Compared to the Con group, the mRNA expression levels of IL-8 and TNF-α declined. IL8 levels in all the treatment groups were decreased and significant in TCM1 and TCM2 (*P* < 0.05). Similarly, the levels of TNF-α were downregulated in the TCM1 (*P* < 0.01) and TCM2 (*P* < 0.05) treatments.

## Discussion

In recent decades, medicinal plants have attracted much attention because of their significant bioactivities such as increasing growth performance, antioxidant activity, antiviral activity, and immunomodulatory activity, which make them suitable as antibiotic replacements (15). TCM as an alternative to antibiotics is receiving more and more attention. Numerous studies have been demonstrated that TCM poses lots of function to livestock and poultry breeding, such as anti-inflammatory, oxidation resistance, resistance to allergy, protect the cardiovascular and antitumor (16, 17). Animal immune response to infection is closely related to immunoglobulin levels. Humoral immunity could help the body prevent infections and diseases, which are mediated by the IgA, IgM, and IgG antibodies (18, 19). It has been found that polysaccharides of traditional Chinese medicine alleviated the decrease of serum IgG concentration in broilers treated with cyclophosphamide (20). Similarly, polysaccharides of traditional Chinese medicine significantly increased the values of immune organ indices and serum IgM and IgG in mice and rats (21, 22). The polysaccharides of traditional Chinese medicine injection increased the content of IgG, IgM, and IgA in weaned piglets (15). We found that adding TCM1 and TCM2 as feed additives in the finishing pigs increased IgA, IgG, and IgM levels compared to the Con group. The complement system restricts viral infections through both classical and alternative pathways (23, 24). They contain numerous small proteins that enhance the ability of antibodies and phagocytic cells to clear microbes and damaged cells in the blood (25, 26). In addition, they also can facilitate inflammation and attack cells infected with pathogens. C3, the body's most abundant complement, is the body's innate immune system central link. It can interact with at least 25 soluble or membrane-bound proteins to activate the complement system through classical pathways, bypass pathways, and lectin pathways (14). In our study, the C3 and C4 in the TCM1 and TCM2 groups were significantly higher than those in the Con group on day 30. The results indicated that TCM1 and TCM2 could enhance immune function in finishing pigs.

Vaccination is often conducted in the pig industry to prevent CSF and PRRS, but there are many factors that can affect its effectiveness. It was described that astragalus polysaccharide and oxymatrine can synergistically improve the immune efficacy of Newcastle disease vaccine in chicken (27). Additionally, Chinese herbal medicine additives can improve the level of antibody against classic swine fever (28). We found that adding TCM1 and TCM2 could increase the levels of antibody against CSFV and PRRSV compared to the Con group. These results indicated that TCM1 and TCM2 could enhance the immune response of finishing pigs by increasing the levels of CSFV and PRRSV antibodies.

In the past few years, a study has shown that many kinds of polysaccharides of TCM have immunomodulatory effects through the TLR4/MyD88/NF-κB signaling pathway (29). TNF-α has the characteristics of a multifunctional pro-inflammatory cytokine with an important role in the pathogenesis of inflammatory diseases in this pathway (30). TNF-α activated phosphorylate IκB and induced its degradation, in parallel with leading to the liberation of NF-κB, and evoking the expression of a variety of genes, which participate in inflammatory responses (31). Our results showed that TCM1 and TCM2 could reduce the expression levels of genes (TLR-4, MyD88, NF-κB, TNF-α, IL6, and IL8) and proteins (IκBα, p-IκBα, p65, and p-p65) to different degrees. Furthermore, this activation was significantly inhibited by TCM1 and TCM2, suggesting that TCM1 and TCM2 exerted important effects resulting in reduced inflammation. Our results showed that TCM1 and TCM2 could act on the NF-κB signaling pathway and reduce the body's inflammatory response, thereby improving the immune performance.

## Conclusion

In summary, the results showed that TCM1 and TCM2 had a significant inhibition of inflammation and low toxicity. These positive effects indicate that TCM1 and TCM2 can be dietary additives for animals to enhance humoral immunity.

## Data availability statement

The original contributions presented in the study are included in the article/supplementary material, further inquiries can be directed to the corresponding author/s.

## Ethics statement

The animal study was reviewed and approved by the Committee for the Care and Use of Experimental Animals, Jiangxi Agricultural University, Jiangxi, China. Written informed consent was obtained from the owners for the participation of their animals in this study.

## Author contributions

XW, JC, and FY contributed to conception and design of the study. YM, AH, and TX organized the database. FW, GZ, and YY performed the statistical analysis. HC wrote the first draft of the manuscript. XG, AH, and TX wrote sections of the manuscript. All authors contributed to manuscript revision, read, and approved the submitted version.

## Funding

The authors declare that this study received funding from Spirit Jinyu Biological Pharmaceutical Co. Ltd. The funder was not involved in the study design, collection, analysis, interpretation of data, the writing of this article, or the decision to submit it for publication.

## Conflict of interest

The authors declare that the research was conducted in the absence of any commercial or financial relationships that could be construed as a potential conflict of interest.

## Publisher's note

All claims expressed in this article are solely those of the authors and do not necessarily represent those of their affiliated organizations, or those of the publisher, the editors and the reviewers. Any product that may be evaluated in this article, or claim that may be made by its manufacturer, is not guaranteed or endorsed by the publisher.
